# Elective Hardware Removal After Long-Term Osseous Integration: A Case Report

**DOI:** 10.7759/cureus.104743

**Published:** 2026-03-05

**Authors:** Tahlia Casey, Arif Akkok, Asli Akkok, Anthony Horvath

**Affiliations:** 1 Surgery, Ross University School of Medicine, Miramar, USA; 2 Orthopaedic Surgery, Lake Erie College of Osteopathic Medicine, Elmira, USA; 3 General Surgery, CUNY School of Medicine, New York, USA; 4 Orthopaedic Surgery, St. John's Episcopal Hospital, New York, USA

**Keywords:** conversion total knee arthroplasty, hardware removal, osseous integration, post-traumatic osteoarthritis, tibial plateau fracture, total knee arthroplasty

## Abstract

Late orthopedic hardware removal following fracture union is most commonly indicated in the setting of post-traumatic osteoarthritis (PTOA), whereas elective removal in asymptomatic patients remains controversial. We present the case of a 53-year-old male with a history of tibial plateau open reduction and internal fixation (ORIF) over a decade prior, who developed severe PTOA requiring total knee arthroplasty (TKA). PTOA is a known long-term complication of tibial plateau fractures, occurring in up to 44% of cases and approximately 5% of patients.Preoperative imaging demonstrated significant osseous overgrowth surrounding the hardware, and operative planning was further complicated by the lack of prior medical records.

Intraoperative findings displayed extensive osseous integration. Following successful removal of periarticular screws, the proximal plate necessitated controlled mechanical fatigue and transection to permit safe extraction while preserving cortical stability. Partial hardware removal facilitated preparation for future arthroplasty while minimizing the risk of neurovascular injury or iatrogenic fracture. This case highlights the technical challenges of delayed hardware removal and supports consideration of earlier elective removal in select patients as a way to mitigate the risks associated with complex late salvage procedures.

## Introduction

Tibial plateau fractures, whether resulting from low-energy mechanisms in osteoporotic bone or high-energy traumatic injuries, often involve disruption of the articular surface. Due to their proximity to the tibiofemoral joint, these fractures carry a significant risk for long-term complications, including joint instability, post-traumatic osteoarthritis (PTOA), and even chronic osteomyelitis. Precise classification of tibial plateau fractures is essential for guiding management and prognostication. The Schatzker classification system remains the most commonly utilized framework for stratifying tibial plateau fractures based on the degree of articular involvement and fracture pattern [1].

The management of tibial fractures depends on the fracture location and severity. While the gold standard treatment of diaphyseal tibial shaft fractures involves intramedullary nailing, tibial plateau fractures typically require open reduction and internal fixation (ORIF) to restore joint congruity and maintain mechanical alignment [1,2]. The use of locking plate constructs has become the standard of care for these injuries, allowing stable fixation, preservation of the articular surface, and promotion of cortical union while reducing the risk of misalignment and subsequent degenerative changes.

Long-term sequelae of tibial plateau fractures may ultimately necessitate total knee arthroplasty (TKA). The presence of retained hardware from prior ORIF introduces substantial technical challenges during arthroplasty, including scar tissue formation, altered anatomy, and potential osseous integration of fixation devices [3]. As a result, surgeons are often confronted with a complex clinical decision: whether to pursue early elective hardware removal to potentially reduce the need for a future TKA or to defer removal and risk a technically demanding procedure years later [4].

## Case presentation

A 53-year-old male with a history of chronic back, wrist, and left knee pain presented for evaluation of progressive left knee osteoarthritis. His surgical history was significant for ORIF of a depressed, comminuted left proximal tibial fracture sustained during a traumatic crush injury. The initial procedure was performed in June 2013, followed by revision fixation in 2014. Based on radiographic findings, the injury was consistent with a possible Schatzker type II tibial plateau fracture [4]. 

**Figure 1:**
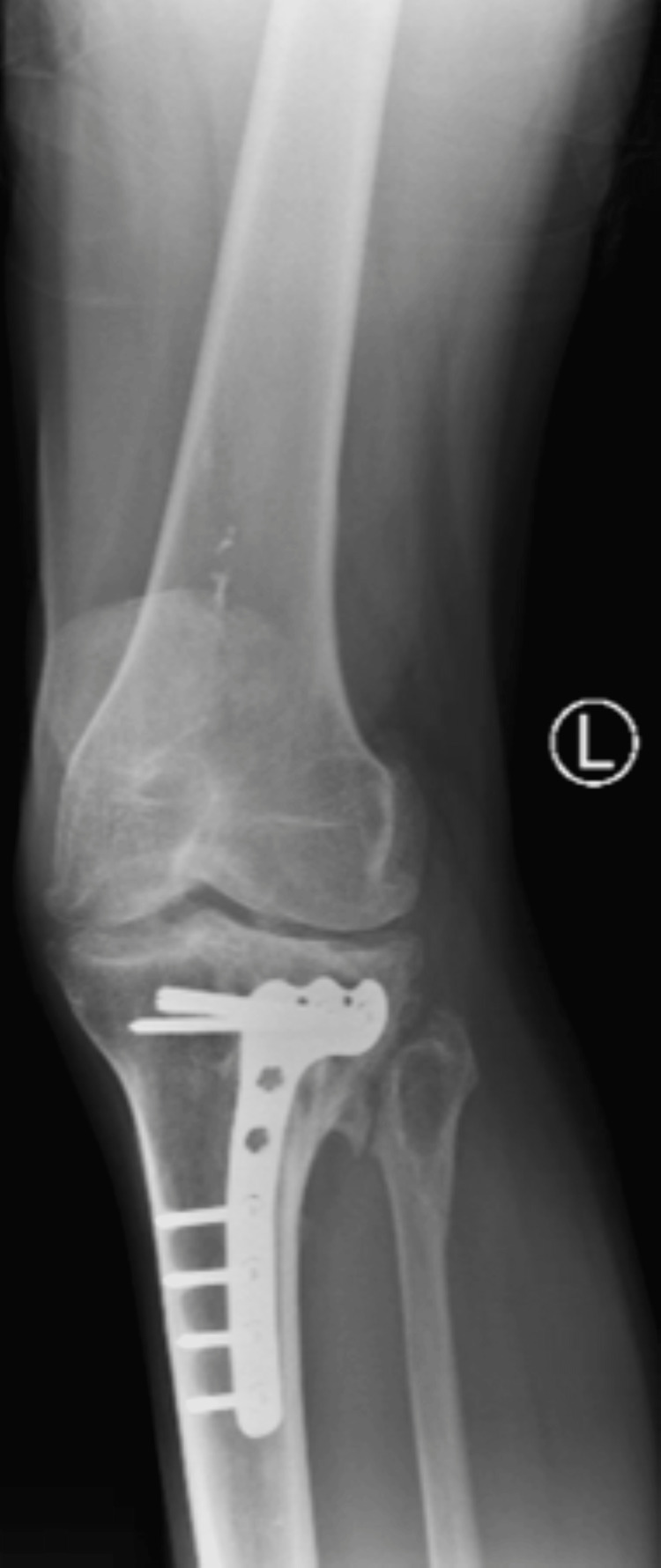
Preoperative radiograph of the left knee demonstrating retained lateral proximal tibial plate-and-screw fixation with advanced medial compartment degenerative changes.

**Figure 2:**
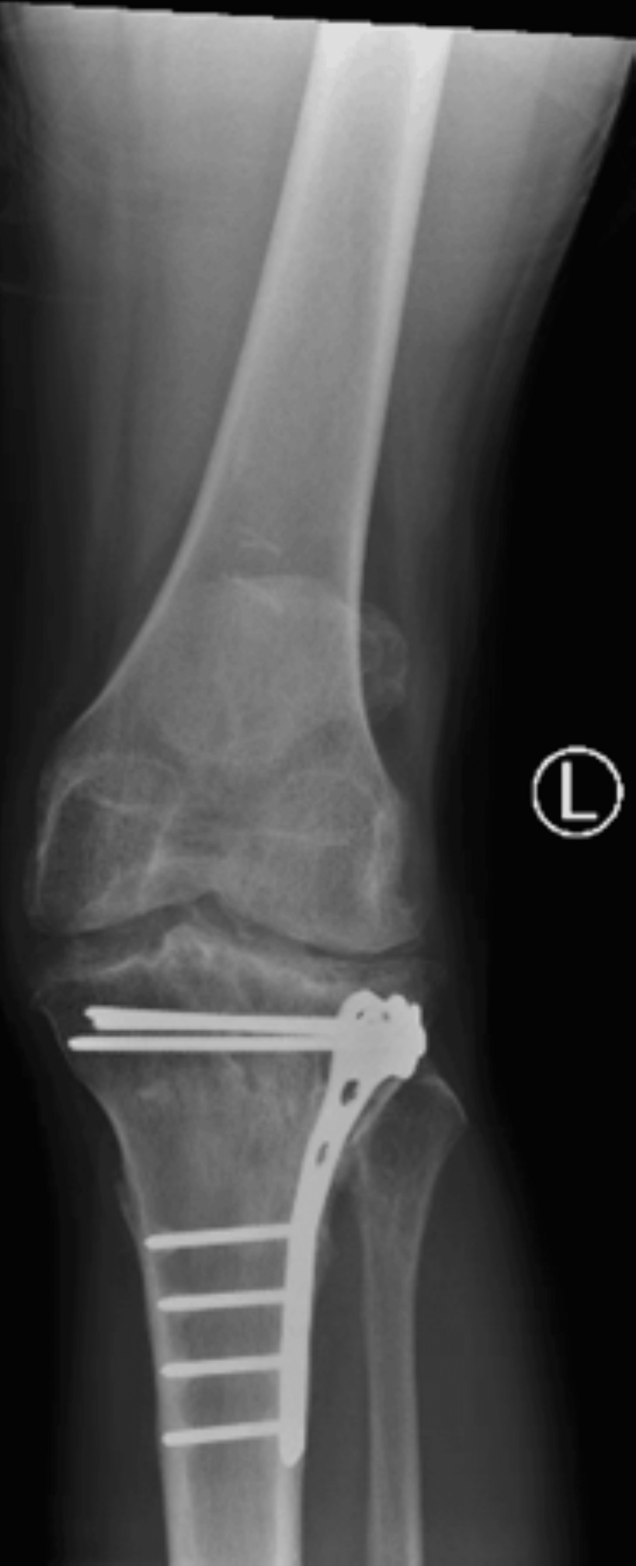
Preoperative radiograph further illustrating the lateral tibial fixation construct with periarticular screws and surrounding osseous remodeling consistent with long-term integration.

In May 2025, the patient presented to the outpatient clinic for evaluation of left knee pain that was refractory to over-the-counter analgesics. Initial conservative management, including physical therapy and medical optimization, failed to provide symptomatic relief. Radiographic evaluation (Figures 1-2) demonstrated marked joint space narrowing consistent with advanced PTOA. Given his radiographic findings and persistent functional limitation, TKA was recommended. However, due to retained hardware from prior ORIF, staged hardware removal was recommended prior to arthroplasty. The patient was counseled regarding the increased surgical risks associated with delayed hardware removal, including iatrogenic fracture, incomplete hardware extraction, prolonged operative time, and extended anesthesia exposure

**Figure 3:**
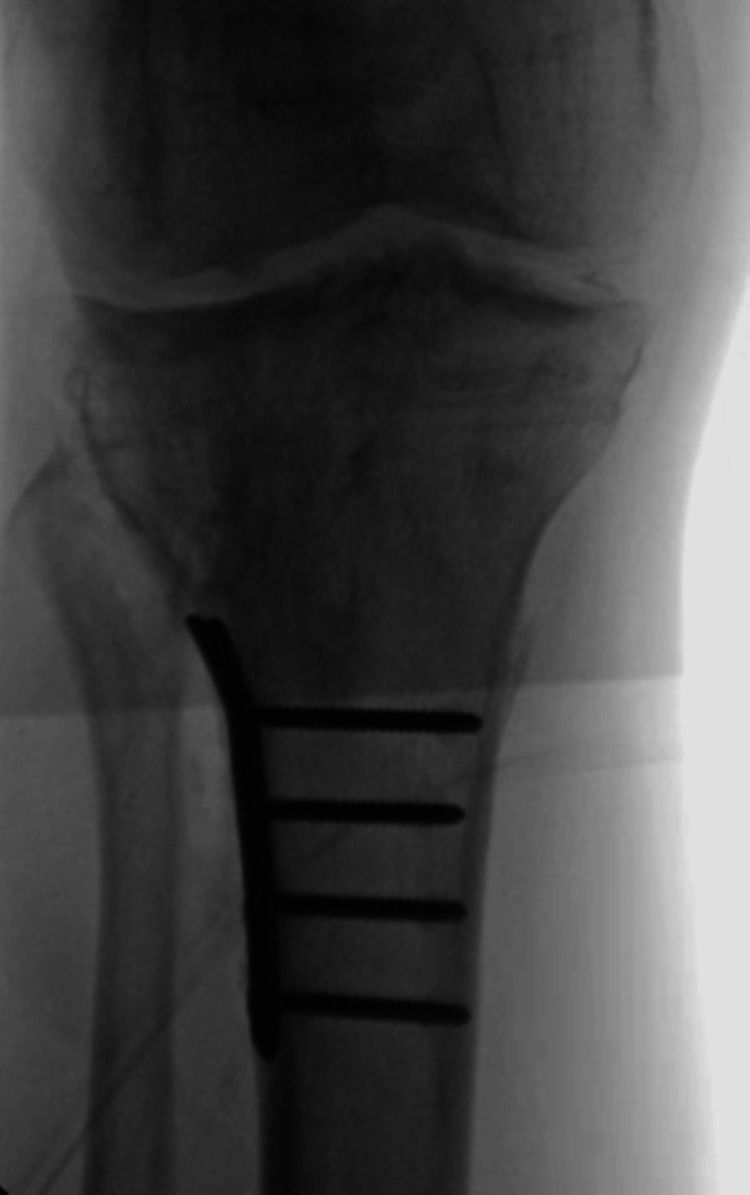
Postoperative radiograph demonstrating removal of proximal periarticular fixation screws with retention of the distal lateral tibial plate segment to preserve cortical stability.

The surgical plan emphasized complete visualization of the fixation plate prior to hardware extraction. The knee was flexed to approximately 120 degrees to optimize exposure of the proximal tibia and facilitate visualization of the periarticular plate. An osteotome was used to carefully remove osseous overgrowth encasing the plate. Initial attempts at screw removal revealed stripped screw heads. A core reamer and mallet were therefore utilized to expose and gain purchase on the periarticular screws, which were extracted using locking pliers. The diaphyseal aspect of the plate was tightly adherent with significant osseous integration. To preserve cortical stability and prevent iatrogenic fracture, the proximal aspect of the plate was carefully elevated using an osteotome. Mechanical fatigue was then applied with reduction pliers, and the plate was transected to allow controlled removal. Postoperative radiographs (Figure 3) demonstrated removal of the proximal periarticular fixation components with retention of the distal diaphyseal plate segment. The proximal rafting screws were no longer visualized. Cortical integrity of the proximal tibia was preserved without evidence of iatrogenic fracture or displacement. Overall alignment was maintained.

Postoperatively, the patient was advised to limit weight-bearing for four weeks to protect cortical integrity. At his two-week follow-up, the surgical site was well-healed, with no evidence of infection or complications. He reported no pain with ambulation. Physical therapy was subsequently initiated to optimize functional status in preparation for TKA. 

## Discussion

Hardware removal more than a decade after tibial plateau ORIF, especially when necessitated by conversion to TKA, constitutes a technically demanding salvage procedure. Extensive osseous integration significantly altered the operative risk profile. Advanced extraction techniques, such as plate transection, were required. These findings highlight how prolonged implant retention can turn a routine hardware removal into a complex reconstructive challenge. 

PTOA is a well-documented sequela of intra-articular tibial plateau fractures, with a significant proportion of patients progressing to TKA. Large-scale studies indicate that patients with tibial plateau fractures have a substantially elevated lifetime risk of TKA compared to the general population [5,6]. Radiographic progression of PTOA has been reported in 22-44% of patients, with 5-7% ultimately requiring TKA within 10 years of the initial injury [7]. This underscores that conversion to arthroplasty is not an uncommon endpoint in this population.

When TKA becomes necessary, retained hardware presents a formidable obstacle. Conversion arthroplasty following tibial plateau fixation is recognized as more demanding than primary TKA and is associated with higher complication rates, longer operative times, and increased risks of infection and stiffness [8,9]. This case exemplifies these findings. Extensive osseous integration necessitated partial retention of the fixation construct to preserve cortical integrity and avoid iatrogenic fracture. This patient with end-stage PTOA underwent higher-risk hardware removal in preparation for TKA that necessitated specialized instrumentation and deliberate mechanical fatigue of the plate, reinforcing the operative risks associated with delayed extraction. 

Management of PTOA depends on activity level, patient age, and present comorbidities. The decision to retain hardware in asymptomatic patients warrants careful consideration, given the documented long-term likelihood of arthroplasty after tibial plateau fractures. The decision for hardware removal should not be based on current symptoms alone [10]. It must weigh the statistically foreseeable need for future TKA against the markedly higher morbidity of late removal [5]. While routine elective removal in all individuals cannot be universally recommended, this case suggests that earlier removal in select patients may mitigate the morbidity associated with late-stage salvage procedures.

## Conclusions

Hardware removal 11 years after tibial plateau ORIF presents a distinct surgical challenge marked by profound osseous integration and altered local anatomy. Although technically feasible, delayed extraction demands a highly strategic approach, specialized instrumentation, and meticulous surgical technique to avoid serious complications. This case underscores how prolonged implant retention can significantly increase procedural complexity and operative risk. In select patients with fracture union, preserved joint function, and minimal comorbid burden, earlier elective hardware removal may warrant consideration to mitigate the challenges associated with late salvage procedures.
